# Reduction of oxytocin plasma levels in borderline personality disorder and normalization induced by psychotherapies

**DOI:** 10.1017/S003329172500042X

**Published:** 2025-03-21

**Authors:** Luisella Bocchio Chiavetto, Daniela Tardito, Chiara Galbiati, Clarissa Ferrari, Mariangela Lanfredi, Laura Pedrini, Antonino Carcione, Ilaria Riccardi, Giuseppe Nicolò, Roberta Rossi, The CLIMAMITHE Study Group

**Affiliations:** 1 Department of Theoretical and Applied Sciences (DiSTA), eCampus University, Novedrate (Como), Italy; 2 Unit of Psychiatry, IRCCS Istituto Centro San Giovanni di Dio Fatebenefratelli, Brescia, Italy.; 3 Fondazione Poliambulanza Istituto Ospedaliero, Brescia, Italy; 4 Third Centre of Cognitive Psychotherapy, Rome, Italy; 5 Italian School of Clinical Cognitivism, Rome, Italy; 6 Department of Human Science, Guglielmo Marconi University, Rome, Italy; 7 Mental Health Department, ASL Roma 5, Colleferro (Rome), Italy

**Keywords:** borderline personality disorder, oxytocin, plasma levels, psychotherapies, metacognitive interpersonal therapy, Structured Clinical Management

## Abstract

**Background:**

Borderline personality disorder (BPD) is a severe mental disorder characterized by emotional dysregulation, impulsive behaviors, and difficulties in interpersonal relationships. Despite the poor understanding of the underlying biological processes, the oxytocin (OXT) system may be involved in and mediate some of BPD’s symptomatic and behavioral aspects. To clarify OXT’s role in BPD, we assessed its plasma levels and modulations induced by psychotherapies in patients.

**Methods:**

Fifty BPD patients and 28 healthy controls (HC) participated in the study; patients were randomly assigned to two psychotherapeutic treatments: metacognitive interpersonal therapy and structured clinical management. Clinical and psychometric measures were assessed, and plasma was collected at baseline (T0) and in patients after 6 (T6) and 12 (T12) months of treatment. OXT was quantified by a radioimmunoassay technique.

**Results:**

BPD patients showed lower plasma OXT at T0 than HC (*p* = 0.002), and a correlation was observed (*r* = −0.36, *p* = 0.017) between low OXT concentrations and high Attachment Style Questionnaire – Italian Version–Preoccupation with Relationships subscale scores. OXT changed significantly over time in patients (*p* = 0.049) with an increase particularly evident from baseline to T6 (*p* = 0.022), without significant difference between treatment groups. OXT changes (T0 − T12) inversely correlated with symptom improvement as changes in the Zanarini Rating Scale for borderline personality disorder (*r* = 0.387, *p* = 0.006) and the Difficulties in Emotion Regulation Scale (*r* = 0.387, *p* = 0.005) scores during treatment.

**Conclusions:**

OXT alteration in BPD patients and the regularizing effect of long-term psychotherapies support an involvement of the OXT system in the disease and in treatment impact. More research is needed to fully understand the underlying causal mechanisms linking OXT with pathogenesis and psychotherapy outcomes.

## Introduction

Borderline personality disorder (BPD) is a severe mental disorder characterized by emotional dysregulation, impulsive behaviors, and difficulties in interpersonal relationships. Interpersonal problems are one of the core symptoms of BPD, described in the DSM-5 BPD criteria as intense and unstable, marked further by abandonment fears and by contrasting feelings between idealization and devaluation. Due to their key role in BPD symptomatology, the domains ‘interpersonal hypersensitivity’ and ‘perceptions of others selectively biased toward negative attributes’ have been integrated as diagnostic criteria into the DSM-5 alternative model of personality disorders (American Psychiatric Association, 2013).

It is well known that BPD patients show poor mentalization/metacognitive abilities in terms of difficulties in understanding mental states of themselves and others, reflecting on them, and using this information to manage subjective suffering and interpersonal relationships (Fonagy & Bateman, 2016). Mentalization/metacognitive failures are often described as possible psychological endophenotypes linked to disturbed interpersonal relationships and rejection sensitivity (Gunderson, 2007). Poor interpersonal functioning is related to impaired social cognition, ranging from alterations of rather basic functions, such as facial emotion recognition (Daros, Zakzanis, & Ruocco, 2013) and low metacognitive functions (Fonagy & Bateman, 2016), to more complex functions, such as trust and cooperation (Seres, Unoka, & Kéri, 2009).

Different psychotherapeutic approaches have demonstrated efficacy in treating BPD patients, some with a theoretical basis, while others are ‘generalist’ (Choi-Kain et al., 2017) and represent the first-choice treatment. Few studies have investigated the effect of psychotherapeutic approaches on the brain in terms of functional and structural correlates. In particular, dialectical behavioral therapy, metacognitive interpersonal therapy (MIT), and structured clinical management (SCM) showed an effect on amygdala activation (Goodman et al., 2014; Rossi et al., 2023).

Concerning the biological mechanisms underlying interpersonal dysfunction, a neuropeptide model of BPD suggests that alterations of oxytocin (OXT) regulation may be critical for the disorder (Stanley & Siever, 2010). Impairments in social cognition are also thought to be linked to adverse early childhood experiences and are fundamentally mediated by the OXT system (Müller et al., 2019).

OXT, a peptide hormone that exerts its effects in response to various stimuli, has a predominantly neuromodulatory role by influencing gene expression, synaptic plasticity, and neurotransmission (Dölen, Darvishzadeh, Huang, & Malenka, 2013; Florea et al., 2022; Xiao et al., 2017). It has been shown that central OXT effects vary from the modulation of some neuroendocrine reflexes related to reproduction and childcare, such as labor and breastfeeding (Gimpl & Fahrenholz, 2001; Uvnas-Moberg et al., 2020), to that of complex behaviors, such as response to various stressors, cognition, and social conducts influencing empathy, recognition of facial emotions, and attachment (Ferreira & Osório, 2022; Froemke & Young, 2021; Gimpl & Fahrenholz, 2001; Montag et al., 2020; Scatliffe, Casavant, Vittner, & Cong, 2019). Although the number of OXT axons in forebrain structures is relatively small, the high receptor affinity of OXT and its supposed action on interneurons allow for the rapid modification of neuronal activity, preferentially in the amygdala, a key region involved in approach and avoidance behavior (Brüne, 2016). In particular, OXT may act as a modulator of attention, increasing the importance of social information and promoting a behaviorally appropriate social response mediated by different brain areas and circuits (Froemke & Young, 2021).

The interface between OXT and BPD has yielded interesting findings, although the exact nature of this relationship remains complex and multifaceted (di Giacomo et al., 2024). Some evidence suggests that reduced OXT expression may be a biological mediator of some aspects of the psychogenesis and psychopathology of BPD (Carrasco et al., 2020). OXT may also affect interpersonal hypersensitivity, which is one of the core symptoms of BPD. Indeed, OXT makes people more sensitive to social and interpersonal cues. Due to abnormal prefrontal cortex modulation, low OXT action on the limbic system would cause an abnormally high amygdala activation, leading to interpersonal hypersensitivity, emotional dysregulation, and impulsive behavior (Carrasco et al., 2020). Furthermore, reduced basal levels of OXT cause the amygdala to become more activated in BPD patients, impairing their ability to comprehend social cues and resulting in aberrant behaviors and emotional dysregulation (Perez-Rodriguez, Derish, & New, 2014).

Since patients with psychiatric disorders present several impairments in terms of behavior, social and emotional skills, and cognitive processing, there has been a growing interest in studying how changes in the oxytocinergic system and OXT peripheral levels in blood and CSF can help to understand and treat different psychiatric disorders. Reduced plasma OXT levels may be one neurophysiological correlation between insecure attachment patterns and the emergence of altered inner working models. Such a scenario could be paradigmatic for the psychopathological signs and symptoms associated with BPD and, above all, for the interpersonal difficulties that these patients experience (Brüne, 2016). In this regard, a recent meta-analysis (Ferreira & Osório, 2022) reported a decrease in peripheral OXT in female patients with personality disorders (Bertsch, Schmidinger, Neumann, & Herpertz, 2013; Bomann et al., 2017; Ebert, Edel, Gilbert, & Brüne, 2018; Jobst et al., 2014). Moreover, BPD patients also showed a greater aversive reaction and a trend for greater OXT reduction after a paradigm of sequential social exclusion than healthy subjects (Reinhard et al., 2022). On the other hand, intranasal OXT treatment is able to increase affective empathy and approach motivation in BPD patients and healthy controls (HC) compared with placebo (Domes et al., 2019). In the past, however, some studies reported that the effects of OXT administration can be limited by individual features and/or situations and can elicit responses not always behaviorally appropriate or uniform (Bartz et al., 2011; Shamay-Tsoory, Aharon-Peretz, & Perry, 2009), thus describing an even more complex picture of OXT’s functions in relation to interpersonal and social cognition (Debbané, 2018).

No data are available to date on possible effects induced by psychotherapies in BPD patients on OXT levels and their association with symptom improvement.

Based on that evidence, we aimed to assess plasma OXT in BPD patients before and during two different psychotherapeutic protocols and to correlate the neuropeptide levels with symptom improvement during treatment, clinical characteristics, and childhood stress exposure.

## Materials and methods

Patients and healthy volunteers as control samples (Healthy Controls, HC) were enrolled in the framework of the CLIMAMITHE study (ClinicalTrials.org: NCT02370316), a randomized clinical trial on the effect of psychotherapy on clinical and neuroimaging outcomes. The full description of the clinical trial protocol and its results has been provided elsewhere (Magni et al., 2019; Rossi et al., 2023). The CLIMAMITHE protocol was approved by the ethical committee of the coordinating center (Protocol No. 67/2014). In the present study, we included all BPD and HC for whom plasma samples were available.

The inclusion criteria for the patients were age 18–45, a diagnosis of BPD (DSM-IV-TR), and signed informed consent. The exclusion criteria were a lifetime diagnosis of schizophrenia, schizoaffective disorder, substance abuse or dependence in the 3 months before the enrolment, bipolar disorder, organic mental syndromes, dementia or cognitive impairment, and relevant neurological signs. Furthermore, pregnant or lactating women and patients receiving concurrent psychotherapy were excluded.

HC underwent the same baseline evaluations as BPD patients. The exclusion criteria were any cognitive impairment or psychiatric/neurologic condition, including alcohol/substance abuse.

From the CLIMAMITHE sample, a subgroup of 50 patients and 28 HC subjects at the beginning of the study accepted to undergo the blood sampling for the OXT analyses. Blood samples from the patients were collected at baseline (T0), after 6 months of treatment (T6), and at a follow-up visit 1 year after the start of treatment (T12), and samples from HC were collected only at T0. Since, among the 50 patients enrolled, some discontinued the clinical intervention, blood samples were collected from only 41 patients at T6 and 35 at T12.

### Clinical assessment

The diagnosis was confirmed using the Structured Clinical Interview for DSM-IV-TR disorders I and II (First, Spitzel, Gibbon, & Williams, 1995; 1994). Patients and HC underwent a multidimensional evaluation assessing various clinical features by scoring emotional dysregulation with the Difficulties in Emotion Regulation Scale (DERS) (Gratz & Roemer, 2004), BPD symptomatology with the Zanarini Rating Scale for borderline personality disorder (ZAN-BPD) (Zanarini et al., 2003), and interpersonal functioning with the Inventory of Interpersonal Problems (IIP) (Pilkonis, Kim, Proietti, & Barkham, 1996). At the baseline, we also evaluated the attachment style by the Attachment Style Questionnaire – Italian Version (ASQ) score (Fossati et al., 2003) and the childhood traumatic experiences by the Childhood Trauma Questionnaire (CTQ) score (Bernstein & Fink, 1998).

### Interventions

Patients were randomly assigned to two intervention groups: MIT (Carcione, Nicolo, & Semerari, 2021) and SCM (Bateman & Fonagy, 2009). Briefly, MIT is manualized and conceptualized to target general psychopathology of personality and is aimed to increase metacognitive abilities following a goal hierarchy: 1) the assessment of symptoms, mental state, metacognitive functions, interpersonal problems, and emotion regulation skills; 2) intervention on the aspects that interfere with therapeutic alliance and/or threaten the patient’s life (in particular, self-harming/suicidal behaviors); 3) intervention on symptoms that cause suffering to the patient; and 4) intervention to promote integration of different mental states. SCM is an evidence-based intervention that reflects the ‘best general psychiatric treatment for BPD’ and is to be used by ‘generalist mental health clinicians’ with minimal additional training. SCM is tailored to BPD symptomatology and employs a supportive approach with case management and advocacy support. In this approach, psychoeducation, problem-solving, explicit safety planning, medication review, and assertive follow-up if appointments are missed are the core elements.

Both treatments consist of a 50-min weekly individual session and a 90-min group session (manualized metacognitive skill training in MIT and a problem-solving group in SCM) that cover a period of about 6 months during the year of treatment.

When needed, BPD patients received pharmacological treatment prescribed following a standardized protocol based on most relevant pharmacological guidelines. Fourteen patients were drug-free at the study baseline (T0). During the whole trial, a substantial stability of the drug therapy assumption was maintained.

Among the 50 patients enrolled for the study, 26 underwent the MIT and 24 the SCM interventions.

### Plasma collection and OXT dosage

Peripheral venous blood samples were collected in the morning, after an overnight fast, in EDTA tubes. Blood samples were kept on ice until plasma separation, which occurred by centrifugation at 1,680 g for 15 min at 4°C. Plasma samples were aliquoted and stored at −80°C until the time of analysis.

Plasma OXT concentrations were quantified by radioimmunoassay (RIAgnosis, Munich, Germany). In brief, for each sample. 300 μl of plasma was extracted using LiChroprep^®^ Si60 (Merck) heat-activated at 690°C for 3 h. A total of 20 mg of LiChroprep^®^ Si60 in 1 ml distilled water was added to each sample, mixed for 30 min, washed twice with distilled water and 0.01 mol/L HCl, and eluded with 60% acetone. A total of 50 μl of assay buffer was then added to the extract followed by 50 μl antibody against OXT (raised in rabbits). After a 60-min preincubation interval, a 10 μl 125I-labeled tracer (PerkinElmer, USA) was added, and samples were allowed to incubate for 3 days at 4°C. Unbound radioactivity was precipitated by activated charcoal (Sigma–Aldrich, St Louis, MO, USA). Under these conditions, an average of 50% of total counts are bound with <5% non-specific binding. The detection limit is in the 0.1–0.5 pg/sample range, depending on the age of the tracer, with typical displacements of 20%–25% at 2 pg, 60%–70% at 8 pg, and 90% at 32 pg of standard neuropeptide. Cross-reactivity with arginine vasopressin (AVP), ring moieties and terminal tripeptides of both OXT and AVP, and a wide variety of peptides comprising 3 (α-melanocyte-stimulating hormone) up to 41 (corticotropin-releasing factor) amino acids are <0.7% throughout. The intra- and interassay variabilities were <10%. Plasma samples were analyzed in different batches; however, all samples from an individual at different time points and matched controls were assayed in the same batch. Serial dilutions of plasma samples containing high levels of endogenous OXT run strictly parallel to the standard curve indicating immuno-identity.

### Statistical analysis

Descriptive statistics were presented in terms of mean and standard deviation (SD) for continuous variables or in terms of frequency and percentage for the categorical ones. Group comparisons for continuous variables were performed by *t*-tests (or Mann–Whitney when appropriate), while for categorical variables, by chi-square tests.

Pearson’s *r* coefficient was computed on the patient group to access i) the correlations among OXT plasma levels, IIP, ASQ, DERS, and ZAN-BPD scores at baseline and ii) the correlation of change (T12 − T0) among OXT, DERS, and ZAN-BPD.

For the longitudinal evaluation of the clinical continuous variables, the generalized linear mixed models were applied by setting *time* (three time points: T0, T6, and T12), *group* (MIT vs. SCM) and *time × group interaction* as fixed effects. Post hoc power analyses were performed to ensure the reliability of longitudinal results.

The significance level was set at 0.05 for two-sided tests. The analyses were performed by using the SPSS version 20 software, Jamovi version 2.16.17, and R statistical software (version 4.4.1 and the package ‘simr’) for the post hoc power analysis of the longitudinal assessments.

## Results

### Baseline evaluations

Demographical and clinical characteristics of the subjects are reported in Table 1 as well as history of alcohol and substance abuse. Patient and control groups were not different for sex distribution and mean age, while scores in interpersonal functioning (IIP) and attachment style (ASQ) scales were significantly higher in patients.

**Table 1.** Demographic characteristics, OXT plasma levels, and clinical assessment at baseline (T0) of the study subjects

	Patients *N* = 50	Controls *N* = 28	*p*-value
Sex (female)	41 (82%)	24 (85.7%)	0.673
History of alcohol abuse (yes vs. no)	24 (48%)	#	#
History of substance abuse (yes vs. no)	29 (58%)	#	#
Psychotropic drug treatment (yes vs. no)	36 (72%)	#	#
	Mean (SD)	Mean (SD)	*p*-value
Age	30.1 (8.1)	28.6 (6.8)	0.402
OXT T0 pg/ml	2.34 (0.48)	3.28 (1.44)	0.002
IIP subscales			
Interpersonal sensitivity	2.5 (0.7)	1.1 (0.6)	<0.001
Interpersonal ambivalence	2.1 (0.8)	0.7 (0.5)	<0.001
Aggression	2.2 (1.0)	0.4 (0.6)	<0.001
Need for social approval	2.4 (0.9)	1.3 (0.7)	<0.001
Lack of sociability	2.1 (1.1)	0.8 (0.6)	<0.001
IIP mean scores	2.2 (0.7)	0.8 (0.5)	<0.001
ASQ subscales			
Confidence	26.3 (6.4)	32.6 (4.0)	<0.001
Discomfort with closeness	40.1 (6.0)	33.2 (5.3)	<0.001
Relationships as secondary	17.7 (6.4)	14.2 (5.8)	0.024
Need for approval	29.4 (6.6)	18.6 (5.0)	<0.001
Preoccupation with relationships	36.2 (6.9)	25.8 (5.5)	<0.001
DERS scores	121.7 (25.6)	62.5 (11.7)	<0.001
ZAN-BPD scores	15.6 (4.6)	1.9 (1.3)	<0.001
CTQ scores	57.2 (16.7)	32.2 (5.3)	<0.001

*Note:* Comparison between patients and controls were assessed by Student’s *t*-test or corresponding non-parametric test for continuous data. ASQ, Attachment Style Questionnaire; CTQ, Childhood Trauma Questionnaire; DERS, Difficulties in Emotion Regulation Scale; IIP, Inventory of Interpersonal Problems; OXT, oxytocin; ZAN-BPD, Zanarini Rating Scale for borderline personality disorder.

As shown in Table 1, baseline OXT plasma levels were significantly lower (*p* = 0.002) in BPD patients (mean = 2.34 pg/ml, SD = 0.48) compared with HC (mean = 3.28 pg/ml, SD = 1.44). No difference in plasma OXT was observed between drug-free patients (*n* = 14) and those undergoing treatment with psychotropic drugs (*p* = 0.74).

Significant differences between patients and HC were also found for IIP and ASQ scores. As expected, no significant differences were found at baseline between the two patient groups (MIT vs. SCM) for all the variables analyzed (data not shown).

### Correlation analysis on the patient group

Correlations among OXT plasma levels and IIP and ASQ subscale scores at baseline were reported in panels A and B of Supplementary Figure S1, respectively. There was no correlation between OXT and the IIP scores: the largest correlation, though not large enough to reach significance, was observed for IIP aggression (*r* = −0.27, *p* = 0.064). Similar results were also found for the correlations between the OXT and ASQ subscale scores, except for the significant negative correlation found for plasma OXT and Preoccupation with Relationships (*r* = −0.36, *p* = 0.017): the lower the OXT levels were, the higher the ASQ Preoccupation with Relationships scores were.

Finally, no significant correlations between OXT levels and ZAN-BPD (*r* = 0.05, *p* = 0.721) or DERS (*r* = −0.04, *p* = 0.787) scale scores were found at baseline. No association was also observed between plasma OXT in patients and CTQ scores (*r* = −0.07, *p* = 0.658).

### Longitudinal evaluation of OXT and clinical features in the patient group

First of all, an analysis of concurrent modifications of plasma OXT and clinical scale scores in patients during treatments was conducted by the correlation of changes. In detail, the change (T12 − T0) in OXT levels resulted significantly correlated to the change (T12 − T0) in DERS (*r* = 0.39, *p* = 0.005) and ZAN-BPD (*r* = 0.39, *p* = 0.006).

OXT plasma levels, as well as DERS and ZAN-BPD scores, in patients during psychotherapy treatment, are reported in Table 2.

**Table 2.** OXT plasma levels, DERS, and ZAN-BPD scores at the three study observation points

	T0 Mean (SD)			T6 Mean (SD)			T12 Mean (SD)		
	All	MIT	SCM	All	MIT	SCM	All	MIT	SCM
OXT (pg/ml)	2.34	2.32	2.35	2.59	2.66	2.53	2.54	2.65	2.43
	(0.48)	(0.46)	(0.50)	(0.53)	(0.48)	(0.58)	(0.58)	(0.41)	(0.70)
DERS	121.7	118.9	124.7	114.4	111.8	116.5	110.5	106.4	114.1
	(25.6)	(25.7)	(25.5)	(28.2)	(28.9)	(28.1)	(26.7)	(28.1)	(25.6)
ZAN-BPD	15.6	14.7	16.5	12.0	13.2	10.9	9.2	10.8	7.7
	(4.6)	(4.8)	(4.2)	(4.3)	(4.3)	(4.1)	(4.0)	(4.0)	(3.5)

*Note:* DERS, Difficulties in Emotion Regulation Scale; MIT, metacognitive interpersonal therapy; OXT, oxytocin; SCM, structured clinical management; ZAN-BPD, Zanarini Rating Scale for borderline personality disorder.

A deep analysis of changes was performed by the mixed model for repeated variables. Post hoc power analysis was performed to assess adequate power for the longitudinal assessment. A power of 0.85 (95% CI: [76.47, 91.35]) was reached for the longitudinal mixed model assessing the change in OXT across the three time points and patient groups (MIT and SCM).

**Figure 1.**
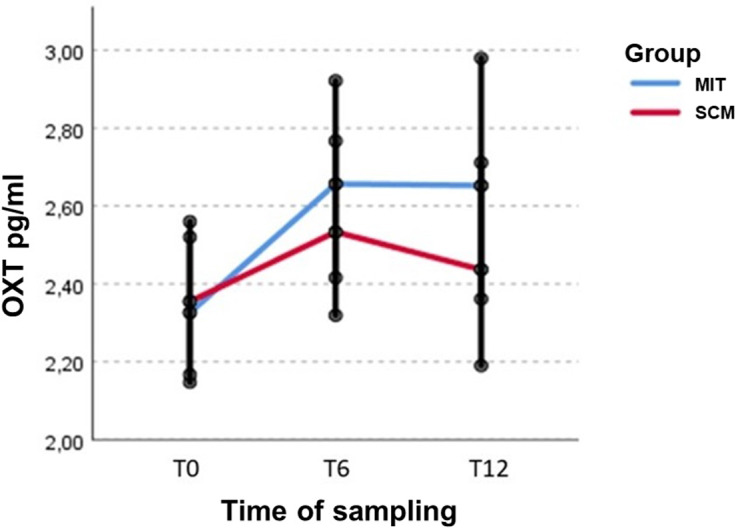
OXT plasma changes in patients during psychotherapies. *Note:* OXT plasma levels are expressed as pg/ml; MIT, metacognitive interpersonal therapy; SCM, structured clinical management.

OXT concentration changed significantly over time (*p* = 0.049), with an increase particularly evident from baseline to T6 (*p* = 0.022). Although changes across time were not significantly different in the two treatment groups, MIT vs. SCM (group × time interaction, *p* = 0.587), patients in the MIT group also show maintenance of higher OXT values at T12 (Figure 1).

Considering the changes in symptomatology, ZAN-BPD scores were reduced during treatment with psychotherapies (*p* < 0.001), while the decrease in DERS scores did not reach significance (*p* = 0.132).

No association was observed between OXT T0 levels and T0–T12 changes in ZAN-BPD and DERS scores (*p* = 0.227 and *p* = 0.910, respectively). No influence of drug treatment was observed on psychotherapies’ effectiveness or OXT levels at T6 and T12.

## Discussion

The results obtained indicate that OXT plasma levels were decreased in BPD patients confirming previous data (Carrasco et al., 2020; Ferreira & Osório, 2022; Mielke et al., 2023). At baseline, no correlation was observed between OXT in patients and BPD symptomatology or emotional dysregulation, as measured by ZAN-BPD and DERS, respectively; however, an inverse correlation was observed with the ASQ Preoccupation with Relationships score, suggesting that this attachment behavior could be linked to the neuropeptide alterations. In this regard, several pieces of evidence indicate that OXT may play a role in attachment (Buchheim & Diamond, 2018; Sharma, Gonda, Dome, & Tarazi, 2020), and recent studies have shown that exogenously administered OXT can affect attachment insecurity (Bernaerts et al., 2017; K. Zhang et al., 2021).

No correlations have been observed between childhood adversity exposure and OXT plasma levels in our samples. These data are in contrast to those of Mielke et al. (2023) reporting an association between plasma OXT and adverse childhood experiences in a wide sample across a large age span (12–50 years) of female individuals with BPD features. Differences in age and clinical characteristics between the samples might explain this discrepancy in the results.

Moreover, no difference in the neuropeptide levels was observed at baseline between drug-free patients and those in pharmacological treatment, suggesting that the decrease in plasma OXT concentration may not be a consequence of the drug therapies, even if this confounding variable could not be ruled out.

In line with previous studies on plasma samples (Ferreira & Osório, 2022), these data support the hypothesis that dysregulations in the oxytocinergic system may be implicated in the pathogenesis of the disorder. Moreover, a reduction in the expression of the OXT receptor (OXTR) in blood mononuclear cells was observed (Carrasco et al., 2020), while genetic studies reported a possible role of the *OXTR* gene in the susceptibility to develop BPD symptoms, showing interactions with family functioning (Hammen, Bower, & Cole, 2015) and childhood maltreatment (Flasbeck, Moser, Kumsta, & Brüne, 2018; M. Zhang et al., 2020).

Of note, here we also report for the first time that two different kinds of psychotherapies are able to increase plasma OXT levels that, in turn, are correlated with symptom improvement, suggesting that these treatments may act biologically on the normalization of the oxytocinergic system. We could hypothesize that psychotherapies might modulate the level of OXT through the creation of therapeutical relationships and alliances and the modulation of attachment relationships. In the CLIMAMITHE clinical trial (Rossi et al., 2023), in which the samples of the present study were collected, MIT and SCM showed an effect in reducing right amygdala activation by viewing emotion stimuli, one of the neural features that has been linked to emotion dysregulation in BPD patients (Schmahl et al., 2014). Considering the present results, we can speculate that the difference in amygdala functionality and the psychotherapy’s effects may be mediated by OXT expression. Indeed, in rodent models, it was shown that oxytocinergic projections to the amygdala are crucial for the discrimination of emotional states(Ferretti et al., 2019), while higher endogenous OXT plasma levels in humans are associated with reduced central amygdala volume and blood oxygen level-dependent activity in response to aversive stimuli (Lancaster et al., 2018). In parallel, intranasal OXT treatment may reduce amygdala hyperactivation in BPD patients (Lischke et al., 2017).

The biological correlates of the efficacy of psychotherapy is still an open and scarcely explored question in biomedical research. Several pieces of evidence indicate that hormones, particularly OXT, may play a role in the mechanism underlying successful treatments (Fischer & Zilcha-Mano, 2022). Most research on the relationship between neuroendocrine markers and psychotherapies has focused on major depression and depressive symptoms, which often co-occur with BPD (Paris, 2018), and a correlation between low OXT plasma levels and diminished symptom improvement after a cognitive behavioral treatment program was observed (Jobst et al., 2018). Moreover, an association was observed between interpersonal difficulties and a lower reduction in depressive symptoms with a lower OXT synchrony between patients and therapists during psychodynamic psychotherapy (Zilcha-Mano, Goldstein, Dolev-Amit, & Shamay-Tsoory, 2021). Recent studies also indicated that absolute salivary OXT reactivity in depressed patients is associated with a larger improvement during a brief psychodynamic treatment (Atzil-Slonim et al., 2022) and that salivary OXT response in therapists may mediate psychotherapy outcome (Fisher et al., 2023). These data suggest that endogenous OXT release may also play a role in synchronization between patients and psychotherapists, which is beneficial for treatment outcomes, therapeutic alliance, and empathy perceived by patients (Palmieri, Pick, Grossman-Giron, & Tzur Bitan, 2021). Regarding BPD patients, a further study has indicated that human- and animal-assisted skills training may increase salivary OXT even if not significantly in a small sample (Plett, Flasbeck, & Brüne, 2023).

Considering the key role of OXT in human interactions (Rigney, De Vries, Petrulis, & Young, 2022), we cannot rule out that the baseline low OXT levels in BPD patients are an epiphenomenon and reflect the poor social functioning and social isolation rather than an illness mechanism, while changes after psychotherapies are a consequence of social functioning improvement rather than direct treatment neurobiological effects.

Our data on the OXT alteration in BPD patients and the regularizing effect of long-term psychotherapies support the involvement of this hormonal system in the pathology and the positive impact of treatments here employed. The fact that psychotherapies can stimulate OXT endogenous release suggests that they may enhance social interactions and emotional regulation in individuals with BPD through a mechanism that might involve amygdala function regulation.

Furthermore, the rise in OXT following psychotherapies here reported may play a role in managing some of the core symptoms of BPD, such as reducing impulsivity and decreasing self-destructive behaviors (Chang et al., 2024; Diaz-Marsá et al., 2024; Mürner-Lavanchy et al., 2024).

It is important to note, however, that the relationship between OXT, pathogenesis, and psychotherapy outcomes in BPD is complex, and more research is needed to fully understand the underlying causal mechanisms.

This study has several limitations that should be considered when interpreting the results. First, our observations were limited to peripheral phenomena, and we do not have direct information on OXT regulation within the brain. This restricts our ability to draw conclusions about the central mechanisms of OXT action and its direct effects on neural processes. Second, the absence of a placebo control group or a non-treated group in our experimental design limits the possibility of assessing the specificity of the observed effects. In this regard, it has to be considered that a relationship between OXT and placebo effects in psychiatric patients has been observed and different evidence suggests that it could be mediated by social facilitation (Itskovich, Bowling, Garner, & Parker, 2022). Third, the correlation observed between OXT plasma levels and Preoccupation with Relationships ASQ scores should be considered only an explorative result that has to be replicated in larger samples, since no correction for multiple testing has been applied. Finally, the limited sample size does not allow us to delve deeper into the associations of baseline OXT levels with specific BPD symptoms and could limit the analysis of possible childhood trauma correlations.

## Conclusion

The study data support the role of OXT alteration in BPD pathology and in the normalizing effects of long-term psychotherapies, which underlies the positive impact of these treatments. In this context, the regulation of OXT expression may represent a biological correlate of treatment impact and/or mediate the establishment of the therapeutic relationship. However, more research is needed to fully understand the underlying causal mechanisms that link OXT with the disease pathogenesis and psychotherapy outcomes.

## Supporting information

Bocchio Chiavetto et al. supplementary material Bocchio Chiavetto et al. supplementary material

## Supplementary material

The supplementary material for this article can be found at http://doi.org/10.1017/S003329172500042X.
