# Supplementary figures and images for: Reduction of oxytocin plasma levels in borderline personality disorder and normalization induced by psychotherapies
Source: Psychol Med. 2025 Mar 21;55:e92. doi: 10.1017/S003329172500042X (PMC12080657; doi:10.1017/S003329172500042X)

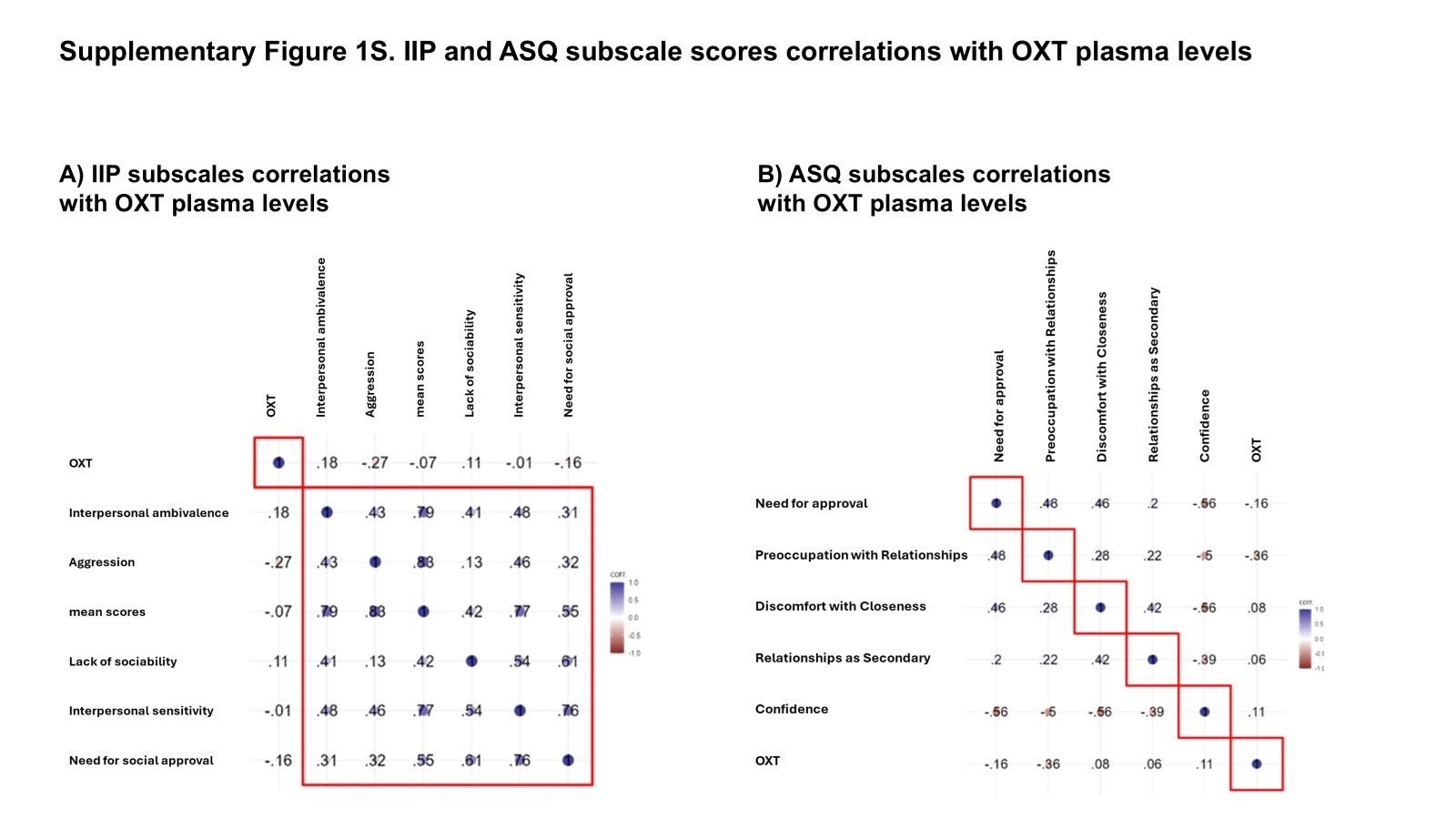

Supplement: Bocchio Chiavetto et al. supplementary material [file S003329172500042Xsup001.zip › S003329172500042Xsup001/Suppl Figure 1.jpg]
